# Segregated fronto‐cortical and midbrain connections in the mouse and their relation to approach and avoidance orienting behaviors

**DOI:** 10.1002/cne.24186

**Published:** 2017-03-20

**Authors:** Michael Anthony Savage, Richard McQuade, Alexander Thiele

**Affiliations:** ^1^ Institute of NeuroscienceNewcastle UniversityNewcastle Upon TyneTyne and WearNE2 4HHUnited Kingdom

**Keywords:** approach behaviors, avoidance behaviors, cingulate area, motor cortex area 2, RRID:SCR_013672, RRID:SCR_013672, superior colliculus

## Abstract

The orchestration of orienting behaviors requires the interaction of many cortical and subcortical areas, for example the superior colliculus (SC), as well as prefrontal areas responsible for top–down control. Orienting involves different behaviors, such as approach and avoidance. In the rat, these behaviors are at least partially mapped onto different SC subdomains, the lateral (SCl) and medial (SCm), respectively. To delineate the circuitry involved in the two types of orienting behavior in mice, we injected retrograde tracer into the intermediate and deep layers of the SCm and SCl, and thereby determined the main input structures to these subdomains. Overall the SCm receives larger numbers of afferents compared to the SCl. The prefrontal cingulate area (Cg), visual, oculomotor, and auditory areas provide strong input to the SCm, while prefrontal motor area 2 (M2), and somatosensory areas provide strong input to the SCl. The prefrontal areas Cg and M2 in turn connect to different cortical and subcortical areas, as determined by anterograde tract tracing. Even though connectivity pattern often overlap, our labeling approaches identified segregated neural circuits involving SCm, Cg, secondary visual cortices, auditory areas, and the dysgranular retrospenial cortex likely to be involved in avoidance behaviors. Conversely, SCl, M2, somatosensory cortex, and the granular retrospenial cortex comprise a network likely involved in approach/appetitive behaviors.

## 1 Introduction

The superior colliculus (SC) is a multimodal sensory‐motor midbrain structure, involved in visual, auditory, and somatosensory triggered orienting (Meredith, Wallace, & Stein, 1992; Stein, 1981; Thiele, Rübsamen, & Hoffmann, 1996; Wallace, Meredith, & Stein, 1993; Westby, Keay, Redgrave, Dean, & Bannister, 1990). In most species, the spatial representation of sensory inputs is aligned to the retinotopic organization of the superficial layers where the central or frontal field/space is represented in the anterior SC, the upper visual hemi‐field in the medial SC, and the lower visual hemi‐field in the lateral SC (Drager & Hubel, 1976; Goldberg & Wurtz, 1972; Meredith & Stein, 1990; Thiele, Vogelsang, & Hoffmann, 1991). Multimodal sensory processing occurs in the intermediate and lower layers where sensory neurons are intermixed with sensory‐motor responses coding for eye (Wurtz & Albano, 1980), head (Harris, 1980), pinnae (Stein & Clamann, 1981), and whisker movements (Bezdudnaya & Castro‐Alamancos, 2014). In primates, electrical microstimulation in intermediate and deep layers of the SC results in defined saccadic eye‐movements, with endpoints in the visual receptive field locations of the stimulation sites (Stryker & Schiller, 1975). This suggests that sensorimotor integration in the SC invariably triggers orienting responses toward the object of interest. However, in rats, stimulation of the SC can elicit orienting responses toward the visual field representation at the stimulation site, and it can result in defensive behaviors such as freezing, or orienting movements away from the visual field region (Dean, Mitchell, & Redgrave, 1988; Dean, Redgrave, & Westby, 1989). These different types of behavior are, at least to some extent, mediated by two separate output pathways from the intermediate and deep layers of the SC. The crossed descending tecto‐reticulo‐spinal projection, which preferentially arises from the lateral SC (Redgrave, Odekunle, & Dean, 1986), is speculated to be involved in approach movements toward novel stimuli. Whereas the uncrossed ipsilateral pathway, of which certain parts arise in the medial SC, is likely involved in avoidance and escape‐like behavior (Westby et al., 1990). This view is in accord with the ecological niches which rodents occupy, where predators most likely appear in the upper visual field, represented medially in the SC, while prey most likely appear in the lower visual field where they can also be detected by the whisker system (Furigo et al., 2010; Westby et al., 1990), which is represented preferentially in the lateral SC (Favaro et al., 2011). In line with this, medial and the lateral parts of the SC in the rat show an anatomical segregation of inputs from subcortical and from cortical sources, which may feed into the avoidance and approach related pathways (Comoli et al., 2012). It is currently unknown whether this distinction holds for the mouse SC, although a recent study has dissected a pathway originating in the intermediate layers of the medial SC. This is involved in defensive behavior, and provides a short latency route through the lateral posterior thalamus to the lateral amygdala (Wei et al., 2015). Beyond the level of the SC, the larger scale cortical and subcortical anatomical networks involved in approach and avoidance behavior in rodents have not been delineated in great detail. In pursuit of this goal, we injected retrograde tracers into the medial or lateral parts of the murine SC (SCm, SCl) to determine their specific input connections. We found that SCl and SCm receive inputs from shared, but also largely distinct sources. The major cortical source of input to SCl originated from motor cortex area 2 (M2) (which in rats has been labeled the frontal orienting field (Erlich, Bialek, & Brody, 2011)), while a major cortical input to SCm arises in the Cingulate Area (Cg). Anterograde injections into M2 and the Cg, reveal output selectivity, which is not limited to the SC. M2 has descending control over a network of areas involved in somatosensation and appetitive behaviors, while Cg has descending control over a network of areas involved in analysis of far sensory processing (vision, audition), and avoidance behaviors.

## 2 Materials and methods

All experiments were carried out in accordance with the European Communities Council Directive RL 2010/63/EC, the U.S. National Institutes of Health Guidelines for the Care and Use of Animals for Experimental Procedures, and the UK Animals Scientific Procedures Act. Animals were housed in standardized cages with ad libitum access to food and water. Surgical protocols were conducted on 18 C57BL6 mice (24–30 g, 3–4 months old, Harlan/Envigo, Blackthorn, Oxfordshire, England).

### 2.1 Surgical protocols

The mice were anesthetized using a mixture of ketamine and medetomidine (0.2 ml 75 mg/kg + 1 mg/kg i.p.) and placed in a stereotactic frame. The dorsal surface of the skull was exposed and prepared for a craniotomy. Craniotomies (0.7 mm) in positions overlying injection sites were made using a microbur (0.7 mm) and a microdrill.

#### 2.1.1 Retrograde tracing

A two‐barreled iontophoresis pipette with a tungsten microelectrode (tip 10–20 microns) (Thiele, Delicato, Roberts, & Gieselmann, 2006) was filled with a 3% (in saline) solution of the retrograde neural tracer fluorogold (FG) (Life Technologies, Warrington, Cheshire, England) (Schmued & Fallon, 1986). The targets were either the SCm (AP ‐3.7 mm, ML 0.2 5 mm, DV 1.5 mm) or the SCl (AP ‐3.7 mm, ML 1.3 mm, DV 2.2 mm). All coordinates were relative to bregma. The pipette was then advanced to the chosen location with a hold current of −500 nA. Once at the target location, the tracer was iontophorized at +500 nA for 30 min (Schmued & Heimer, 1990). After this the current was changed to a hold current of −500nA for removal of the probe.

#### 2.1.2 Anterograde tracing

A calibrated air pressure micropipette was filled with 15% Biotinylated Dextran Amine MW‐10,000 (BDA in saline, Life Technologies, Warrington, Cheshire, England) (Veenman, Reiner, & Honig, 1992). The targets were either the M2 (AP 1.1 mm, ML 0.7 mm, DV 1.5 mm (from brain atlas) or DV 0.6 mm (from brain surface)) or the Cg (AP 1.1 mm, ML 0.25 mm, DV 1.8 mm [from brain atlas], or DV 1.5 mm [from brain surface]). All coordinates were relative to bregma. Once the micropipette was advanced to the target location, a volume of 66 nl was injected over a period of 5 min.

In both protocols (anterograde and retrograde injections), the pipette was left for 20 min after the injections before removing it to allow for optimum diffusion of tracer into the tissue.

After a 3–4 days recovery period, the mice underwent a cardiac perfusion. They were given terminal anesthesia of pentobarbital (0.3 ml 200 mg/ml i.p.). Then they were perfused, with a preliminary injection of 1 ml heparin sulfate (5,000 I.U./ml) (Hayat, 2012), followed by a 4% paraformaldehyde in phosphate buffer solution (PBS) with 20% sucrose for 30 min at 1ml/min (Rosene & Mesulam, 1978). Post perfusion, brains were removed and placed in the paraformaldehyde solution to post‐fix for 24 hrs. After post‐fixing, the brains were cryo‐protected in a 30% sucrose solution for another 24 hrs period.

### 2.2 Histology

#### 2.2.1 Retrograde FG tracing

Coronal free floating sections (40 µm) were taken and placed in 4% PBS. This was followed by an initial autofluorescence quenching step (20 min 1% sodium borohydride wash, a 20‐minute wash with 5 mM Glycine) and PBS washes (3 × 10 min). Sections were then mounted onto microscope slides with a propidium iodide (PI) medium (Vectashield H‐1300, Vectorlabs, Peterborough, Cambridgeshire, England) or a DAPI medium (Vectashield H‐1500, Vectorlabs, Peterborough, Cambridgeshire, England).

#### 2.2.2 Anterograde BDA tracing

Coronal free floating sections (40 µm) were taken and placed in 4% PBS. After an initial autofluorescence quenching step (as for retrograde tracing), sections were incubated for 2 hrs in streptavidin‐Alexa 488 (Life Technologies, Warrington, Cheshire, England) (Wang & Burkhalter, 2007) (1:500 in 1% normal bovine serum, 0.2% triton X, 0.1% gelatine in PBS) at room temperature followed by PBS washes (3 × 10 min). Sections were then mounted onto microscope slides with a DAPI medium (Vectashield H‐1500).

### 2.3 Fluorescence microscopy

For the retrograde experiments with unamplified fluorescence, sections were examined under a fluorescence microscope (Leica DM LB 100T), at an excitation wavelength of 350 nm to illuminate endogenous FG fluorescence. Excitation at 530 nm was utilized to highlight nuclei with the PI staining and co‐locate with the tracer signal. Digital images were acquired using “MicroFire” optics.

Sections from the anterograde tracing, which had undergone immunohistochemical amplification were examined under a fluorescence microscope (Zeiss Axioimager II, Zeiss Zen software RRID:SCR_013672). Projection patterns were visualized with excitation at 500 nm; nuclei counterstains were visualized with either 530 nm excitation (PI) or 350 nm (DAPI). Photo‐merges were taken of stained areas for further qualitative and quantitative analysis using AxioVision software. For illustrative purposes photomicrographs were processed for brightness and contrast and gray‐scaled using Adobe Photoshop CS6.

### 2.4 Contour plots of injection sites

In order to display the extent of our injections, photomicrographs of each injection case were taken for each animals. These were then processed using ImageJ/Fiji (RRID:SCR_002285) to remove background luminance and were thresholded. This was achieved through custom scripts which calculate the thresholding value (*L*_thresh_) according to the following formula:

$$L_{\text{thresh}}=L_{\text{mean}}\left(\text{ROI}\right)+L_{\sigma^{2}}\;\left(\text{ROI}\right)$$

$$L_{\text{thresh}}=L_{\text{mean}}\left(\text{ROI}\right)+L_{\sigma^{2}}\;(\text{ROI})$$

where *L*_mean_ corresponds to the mean luminance across the region of interest (ROI), and $L_{\sigma^{2}}$ corresponds to the variance of the luminance across the ROI. The ROI chosen for the luminance thresholding was taken from nonlabeled regions of the photomicrograph. Thresholding produced a binary image, where values of 1 displayed the extent of tracer injection. From these images, a contour outlining the extent of labeling was produced by demarcating the limits of the binary signal. These contours were then imported into a vector graphics program and transposed onto representative brain atlas slides (Franklin & Paxinos, 2012).

### 2.5 Analysis of tracing data

#### 2.5.1 Retrograde

For quantitative analysis of the retrograde tracing study, images were processed with ImageJ 2 (Schindelin et al., 2012). For this, we wrote scripts which performed a Gaussian Convoluted Background Subtraction (sigma = 20) to remove biological artefacts, and to filter and grayscale the images. ROIs for brain regions were defined and demarcated on nuclear counterstained images (DAPI, PI) using the mouse brain atlas as reference (Franklin & Paxinos, 2012). Images underwent semiautomated cell counting for each injection case. Based on these numbers, we calculated the proportion of cells labeled in any brain area (from all cells labeled across the brain of a given experimental animal), and used these to calculate proportions across our experimental animals. To simplify the presentation and classification we additionally report the labeling extent in 5 categories of connectivity strength, whereby areas with no input to the SC were labeled with a “−,” low (<2.5%) input with “+,” medium (<5%) input with a “++,” high input (5–7.5%) with a “+++,” and very high input (>7.5% of cells labeled (from all cells labeled) as “++++” which are displayed in Table 1.

**Table 1:** Qualitative densities of retrogradely labeled brain areas after injection of fluorogold in the medial and lateral superior colliculus

		SC (m)		SC(l)	
		Ipsi	Contra	Ipsi	Contra
**Cortex**					
***Prefrontal***					
Cg	cingulate cortex	++++	−	+	−
M1 (An)	primary motor cortex (anterior)	−	−	+	−
M2 (An)	secondary motor cortex (anterior)	−	−	++++	−
M2 (Pos)	secondary motor cortex (posterior)	++	−	++++	−
***Sensory***					
Au1	primary auditory cortex	+	−	−	−
RSD	retrosplenial dysgranular cortex	+++	−	−	−
RSG	retrosplenial granular cortex	−	−	+	−
S1BF	primary somatosensory cortex, barrel field	−	−	+++	−
S1FL	primary somatosensory cortex, forelimb region	−	−	+	−
V2L	secondary visual cortex, lateral area	++	−	−	−
V2ML	secondary visual cortex, mediolateral area	++	−	−	−
V2MM	secondary visual cortex, mediomedial area	+++	−	−	−
**Thalamus**					
LPMR	lateral posterior thalamic nucleus, mediorostral part	+	−	−	−
ZID	zona incerta, dorsal part	+	−	++	−
ZIV	zona incerta, ventral part	+++	−	++++	−
**Hypothalamus**					
LH	lateral hypothalamic area	+	−	+	−
VMH	ventromedial hypothalamus	++	−	−	−
**Pretectum**					
PCom	nucelus of the posterior commissure	++	−	+++	+
PT	pretectal area	++	+	−	−
**Midbrain**					
DRV	dorsal raphe nucleus	+	+	−	−
ECIC	external cortex of the inferior colliculus	+++	+	−	−
ll	lateral lemniscus	++	−	++	−
mRt	mesencephalic reticular formation	+	+	++++	++
PAG	periaqueductal gray	+	+	+	+
PBG	parabigeminal nucleus	++	+	−	−
Pn	pontine nuclei	+++	++	−	−
PR	prerubral field	−	−	+	−
SC (l)	superior colliculus (lateral part)	+	+	N/A	+
SC (m)	superior colliculus (medial part)	N/A	−	+++	−
SNR	substantia nigra, reticular part	++	+	++++	++
STh	subthalamic nucleus	+	−	−	−

Relative cell count densities were assigned one of five levels via quantitative assessment of percentage of total cells labeled in each case then averaged across the entire experimental cohort (none “−” 0%, low “+” < 2.5%, medium “++” < 5%, high “+++”<7.5%, and very high“++++” > 7.5%). See methods for more details. Injection sites could not be quantified in this manner due to tracer spread and were therefore marked with N/A.

#### 2.5.2 Anterograde

For representation of the anterograde tracing data in Table 2, the images underwent qualitative visual inspection and were (subjectively) classified into one of five signal strengths, none “−,” low “+,” medium “++,” high “+++,” and very high “++++.” Furthermore, to convey the full range of labeling observed in both the retrograde and anterograde data, a connectivity map was generated.

**Table 2:** Qualitative densities of anterogradely labeled brain areas after injection of BDA in the cingulate area of motor cortex area 2

		M2		Cg	
		Ipsi	Contra	Ipsi	Contra
**Cortex**					
***Association/multimodal***					
Cl	claustrum	+	++	+	++
Ect	ectorhinal cortex	+	+	−	−
M1 (Pos)	primary motor cortex (posterior)	++	−	−	−
M2 (An)	secondary motor cortex (anterior)	++	+	−	−
M2 (Pos)	secondary motor cortex (posterior)	+++	++	++	+
Post	postsubiculum	+	−	−	−
PRh	perirhinal cortex	+	+	−	−
RSD	retrosplenial dysgranular cortex	+++	−	++	−
RSG	retrosplenial granular cortex	+	−	+	−
***Parietal***					
LPtA	lateral parietal association cortex	++	−	−	−
MPtA	medial parietal association cortex	++	−	−	−
***Prefrontal***					
AI	agranular insular cortex	+	−	−	−
Cg1 (An)	cingulate cortex, area 1 (anterior)	+	−	++	−
Cg1 (Pos)	cingulate cortex, area 1 (posterior)	−	−	+++	++
Cg2 (An)	cingulate cortex, area 2 (anterior)	−	−	++	+
DP	dorsal peduncular cortex	+	−	+	−
DTT	dorsal tenia tecta	−	−	++	−
LO	lateral orbital cortex	+++	+	−	−
MO	medial orbital cortex	++	−	++	−
PrL	prelimbic cortex	++	−	++++	−
VO	ventral orbital cortex	+	+	−	−
***Sensory***					
S1BF	primary somatosensory cortex, barrel field	++++	−	−	−
S1FL	primary somatosensory cortex, forelimb region	++	−	−	−
S1HL	primary somatosensory cortex, hindlimb region	++	−	−	−
S1Tr	primary somatosensory cortex, trunk region	+	−	−	−
V1	primary visual cortex	+	−	+	−
V2L	secondary visual cortex, lateral area	++	−	−	−
V2ML	secondary visual cortex, mediolateral area	−	−	+	−
V2MM	secondary visual cortex, mediomedial area	++	−	+	−
**Basal ganglia**					
Cpu (dl)	caudate putamen (striatum), dorsolateral	++	+	−	−
Cpu (dm)	caudate putamen (striatum), dorsomedial	++	−	+++	+
GP	globus pallidus	+	−	−	−
**Basal forebrain**					
AcbC	accumbens nucleus, core	−	−	+	−
HBO	horizontal limb diagonal band	−	−	++	−
LS	lateral septal	−	−	+	+
MS	medial septal	−	−	+	+
VBD	nucleus of the vertical limb of the diagonal band	−	−	+	+
**Thalamus**					
AM	anteromedial thalamic nucleus	++	−	+	−
AVDM	anteroventral thalamic nucleus, dorsomedial part	+	−	+	−
AVVL	anteroventral thalamic nucleus, ventrolateral part	+	−	+	−
CL	centrolateral thalamic nucleus	++	−	++	−
CM	central medial thalamic nucleus	−	−	+	−
DLG	dorsal lateral geniculate nucleus	−	−	+	−
IAD	interanterodorsal thalamic nucleus	−	−	++	+
LDDM	laterodorsal thalamic nucleus, dorsomedial part	++	−	−	−
LDVL	laterodorsal thalamic nucleus, ventrolateral part	++	−	+	−
LHb	lateral habenular nucleus	−	−	++	−
LPMR	lateral posterior thalamic nucleus, mediorostral part	++	−	+	−
LPLR	lateral posterior thalamic nucleus, laterorostral part	+	−	−	−
MDL	mediodorsal thalamic nucleus, lateral part	++	−	+	−
PC	paracentral thalamic nucleus	−	−	+	−
Po	posterior thalamic nuclear group	+	−	−	−
Re	reuniens thalamic nucleus	+	+	++	++
Rt	reticular nucleus (prethalamus)	++	−	++	−
Sub	submedius thalamic nucleus	+	−	+	−
VA	ventral anterior thalamic nucleus	++	−	+++	−
VM	ventromedial thalamic nucleus	++	−	++	−
VL	ventrolateral thalamic nucleus	+	−	−	−
VPM	ventral posteromedial nucleus	+	−	−	−
ZID	zona incerta, dorsal part	++	−	++	−
ZIV	zona incerta, ventral part	++	−	++	−
**Midbrain**					
ECIC	external cortex of the inferior colliculus	−	−	+	−
IP	interpeduncular nucleus	−	−	++	−
MnR	median raphe nucleus	−	−	+	+
mRt	mesencephalic reticular formation	+++	−	++	−
PAG	periaqueductal gray	+	−	++	−
PMnR	paramedian raphe nucleus	−	−	++	−
Pn	pontine nuclei	−	−	++	−
SC (l)	superior colliculus (lateral part)	++++	−	++	−
SC (m)	superior colliculus (medial part)	++	−	+++	−
SNCD	substantia nigra, compact part, dorsal tier	++	−	+	−
SNR	substantia nigra, reticular part	+	−	+	−
STh	subthalamic nucleus	−	−	+	−
**Hypothalamus**					
PLH	peduncular part of lateral hypothalamus	−	−	+	−
**pretectum**					
APT	anterior pretectal nucleus	+	−	+	−
**Amygdala**					
BLA	basolateral amygdaloid nucleus, anterior part	−	−	++	−

Relative percentage area coverage measured in five levels (none “−,” low “+,” medium “++,” high “+++,”and very high “++++”) for anterogradely traced brain regions averaged across the experimental cohort. These measures were assigned via nonquantitative visual assessment.

### 2.6 Quantitative analysis

For both retrograde and anterograde tracing, images were processed with ImageJ 2 software (Schindelin et al., 2012). This entailed Gaussian filtering (sigma = 3.5) to remove acquisition and biological artefacts. Images were then converted to grayscale and background luminance removal and thresholding was conducted to allow for cell counting and fiber stain assessment. This was achieved through custom scripts which calculate the thresholding value (*L*_thresh_) according to the following formula:

$$L_{\text{thresh}}=L_{\text{mean}}\left(\text{ROI}\right)+L_{\sigma^{2}}\;\left(\text{ROI}\right)$$

$$L_{\text{thresh}}=L_{\text{mean}}\left(\text{ROI}\right)+L_{\sigma^{2}}\;(\text{ROI})$$

where $L_{\text{mean}}$ corresponds to the mean luminance across the region of interest (ROI), and $L_{\sigma^{2}}$ corresponds to the variance of the luminance across the ROI. As described previously, ROIs selected for thresholding were placed on areas which had no clearly labeled cells or fibers. ROIs for cell counting and fiber label assessment were defined and demarcated on nuclear counterstained images (DAPI, PI) using the mouse brain atlas as reference (Franklin & Paxinos, 2012). The tracer signals within the ROI were then quantified by automated cell counts/area (retrograde tracing) or percentage area expressing the tracer signal (anterograde tracing). Quantitative analysis of anterograde tracing was restricted to a few areas, namely those where we predicted they would be preferentially involved in avoidance versus approach. Modulation indices were calculated for these areas (see below).

Preferential connectivity of a particular injection site to different ROIs was determined by calculating the modulation index (MI) of connectivity which was calculated as:

$$\text{MI}=\frac{Q\left(\text{ROI}a\right)-Q\left(\text{ROI}b\right)}{Q\left(\text{ROI}a\right)+Q\left(\text{ROI}b\right)}$$

where  Q(ROIa) corresponds to the quantified amount of tracer in a particular region of interest, and Q(ROIb) corresponds to the quantified amount of tracer in a complementary region. A preference in connectivity for ROIa would yield a positive number between 0 and 1, a preference for ROIb would yield a negative number between 0 and 1. The code for all of the analysis is available online (https://github.com/GrimmSnark/Image_analysis_fiji). Significant differences between the MIs for the particular injection site were tested by a Mann–Whitney U test, alpha value = 0.05.

## 3 Results

We injected the retrograde tracer FG iontophoretically into the SCm or SCl, and we injected the anterograde tracer BDA into the two main cortical SC input structures which are assumed to be key structures involved in top–down behavioral control, namely the Cg or M2. We found that the intermediate and deep layers of the SCl and SCm showed a segregation with respect to specific cortical and subcortical afferents. Moreover, Cg and M2 showed equally substantial segregation regarding their projection sites. The specificity of these connections supports the hypothesis that the medial SC and the Cg are involved in avoidance (aversive) behavior, while SCl and M2 are involved in approach (appetitive) behavior. We will first describe the results from the experiments where retrograde tracers have been injected into the SC, and then describe the experiments where anterograde tracers have been injected into M2 and Cg, respectively.

### 3.1 Retrograde tracing

**Figure 1:**
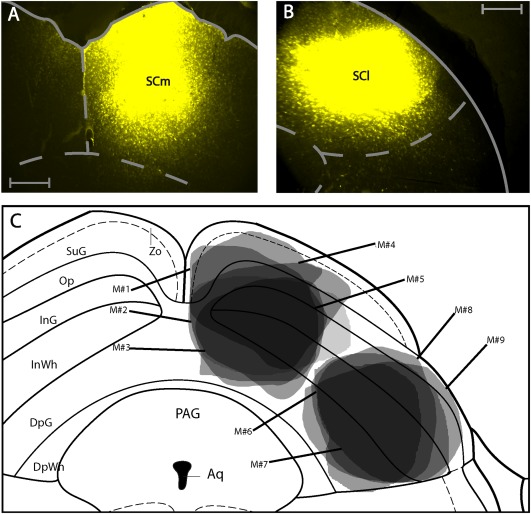
Retrograde tracer injections in the superior colliculus. (a) Photomicrograph of fluorogold injection into the medial superior colliculus. (b) Photomicrograph of fluorogold injection into the lateral superior colliculus. All scale bars equate to 250 µm. (c) Summary of injections. Each shaded area represents the extent of the labeled injection site for both medial and lateral SC conditions. The darker shading indicates overlap of injection volume. Nomenclature in this and all others figures is derived from Franklin, K.B.J. & Paxinos, G. 2012. For abbreviations see list

**Figure 2:**
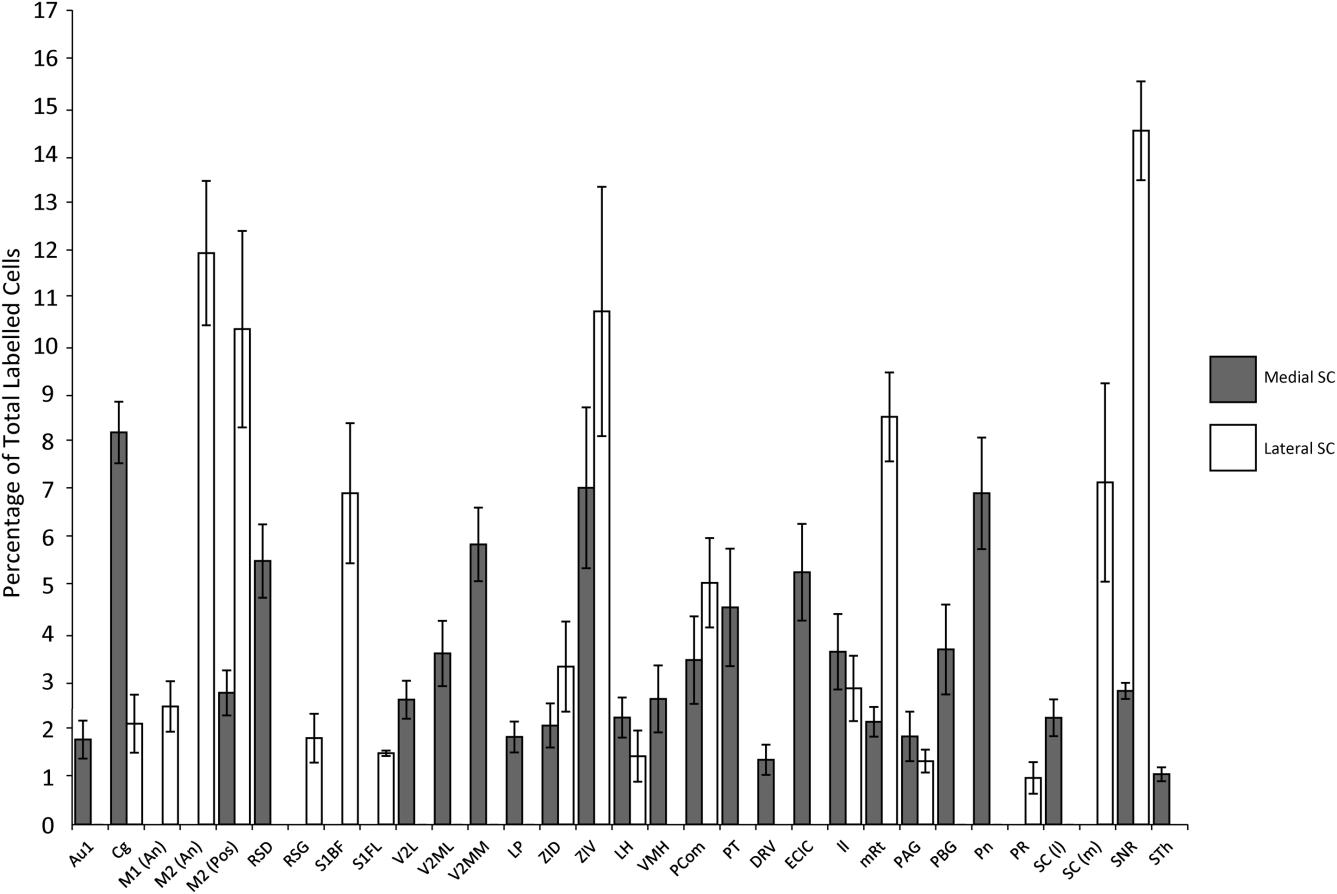
Summary of average percentage of total labeled cells for ipsilateral brain areas after injections of fluorogold into the medial (gray) and lateral (black) superior colliculus. Error bars represent 95% confidence intervals

**Figure 3:**
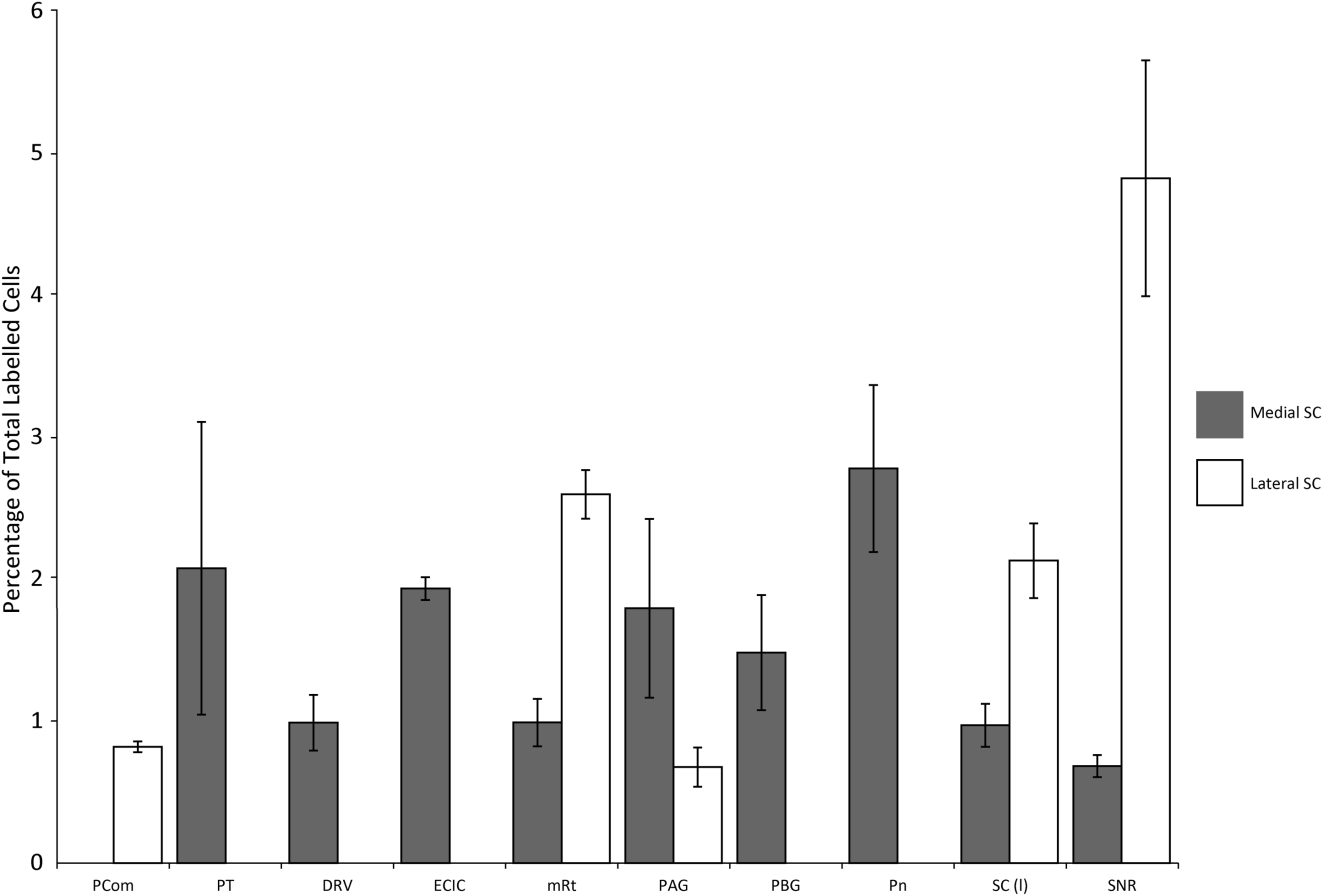
Summary of average percentage of total labeled cells for contralateral brain areas after injections of fluorogold into the medial (gray) and lateral (black) superior colliculus. Error bars represent 95% confidence intervals

We performed five medial and four lateral injections for retrograde tracing in the mouse SC. Local spread of tracer in all of these cases was confined to the target sites in the SC, that is, lateral injections did not spread into medial parts and vice versa. The injections also did not spread into neighboring brain areas such as the periaqueductal gray (PAG) or the mesencephalic reticular formation (mRt) (Figure 1a–c). Retrogradely labeled cells usually arose from areas located ipsilateral to the injection site, but occasionally also from areas contralateral to the injection site. To distinguish these two, we will delineate them by the addition of the terms “ipsilateral”, “contralateral”, and “bilateral”. We will first describe the cortical areas, where retrograde label was found, followed by a description of subcortical areas where retrograde label was identified. We will initially describe those areas that project exclusively to either the SCl or the SCm, followed by a description of areas that project to both SC subdivisions and focus on areas where retrograde label was medium to strong. A complete list of all structures that showed retrograde label after SC injections is given in Table 1 and Figures 2 and 3.

### 3.2 Retrograde labeling in the cortex

Retrogradely labeled cell populations in the neocortex, after injection into the two different subdivision of the SC, were remarkably segregated. As expected, retrogradely labeled cells in the cortex were confined to layer 5b.

**Figure 4:**
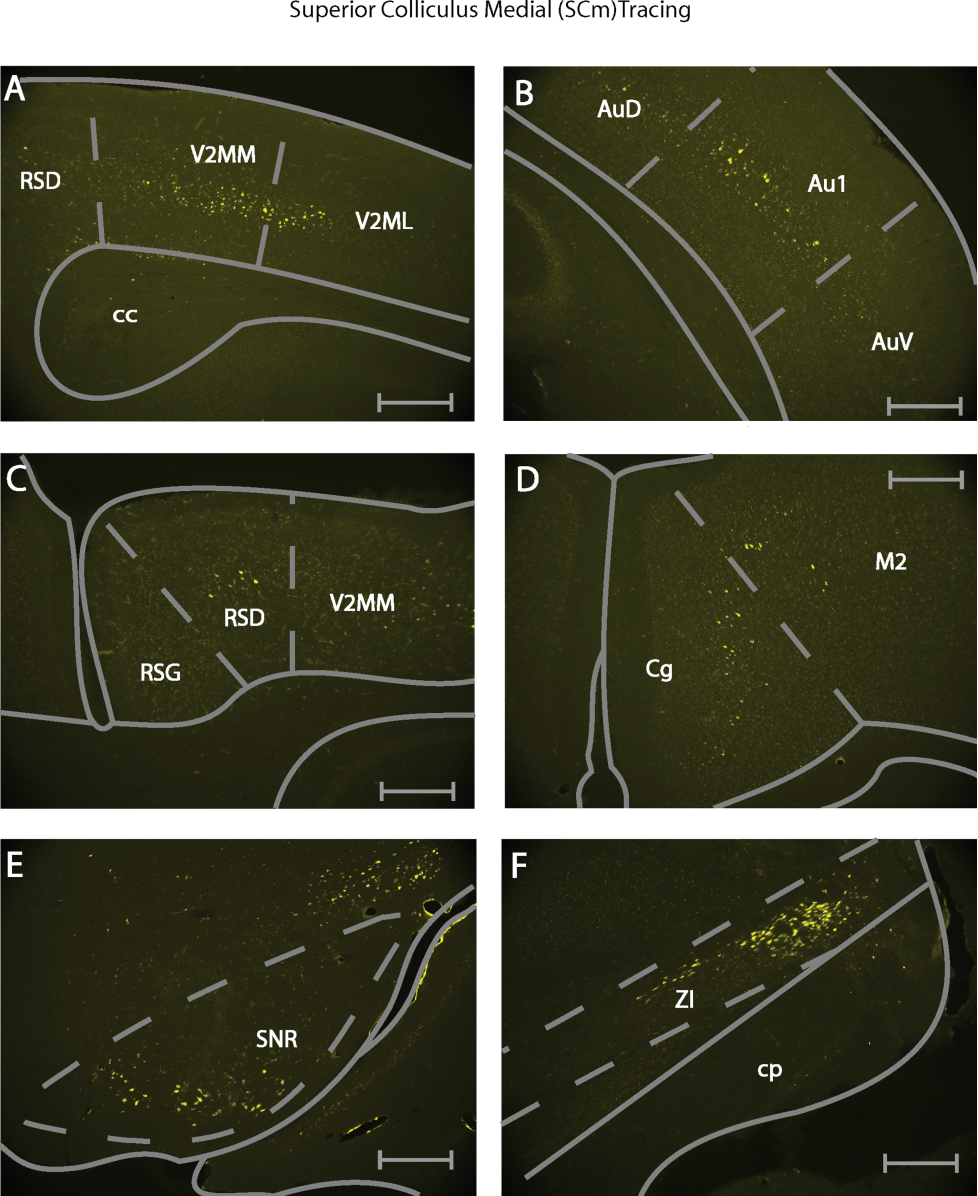
Example photomicrographs of retrogradely labeled brain areas after injection of fluorogold into the medial superior colliculus. (a) Labeling seen in the secondary visual cortex (V2MM/V2ML). (b) Labeling seen in the primary auditory cortex (Au1). (c) Labeling seen in the dysgranular retrospenial cortex (RSD). (d) Labeling seen in the cingulate area (Cg) and motor cortex area 2 (M2). (e) Labeling seen in the ventromedial substantia nigra (SNR[vm]). (f) Labeling seen in the dorsolateral zona incerta (ZI). All scale bars equate to 250 µm

The secondary visual cortex (V2MM, V2ML, V2L, ipsilateral) (Figure 4a), the primary auditory cortex (Au1, ipsilateral) (Figure 4b), as well as the dysgranular portion of the retrosplenial cortex (RSD, ipsilateral) (Figure 4c) showed retrograde labeling only after SCm injections.

**Figure 5:**
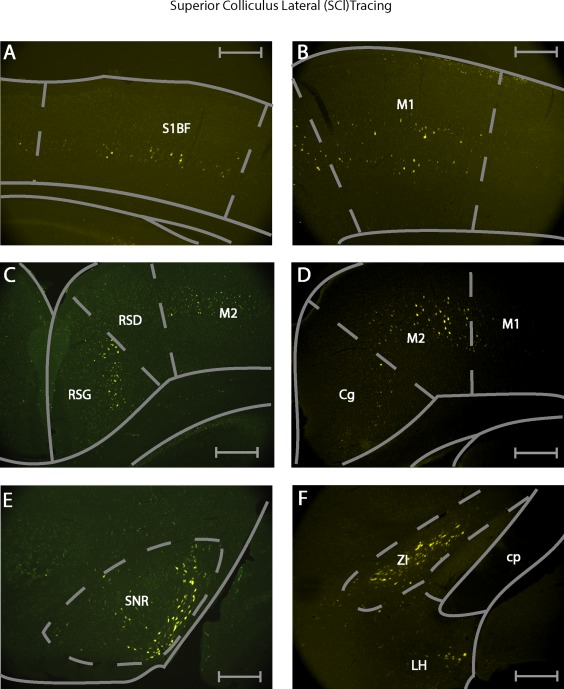
Example photomicrographs of retrogradely labeled brain areas after injection of fluorogold into the lateral superior colliculus. (a) Labeling seen in the primary somatosensory area (S1BF). (b) Labeling seen in the primary motor cortex (M1). (c) Labeling seen in the granular retrospenial cortex (RSG). (d) Labeling seen in the Cg and M2. (e) Labeling seen in the dorsolateral SNR. (f) Labeling seen in the ventromedial ZI. All scale bars equate to 250 µm

Conversely, the somatosensory areas, specifically S1, the barrel field (S1BF, ipsilateral) (Figure 5a), the flank region (S1FL, ipsilateral), the primary motor cortex (M1, ipsilateral) (Figure 5b), as well as the granular portion of the retrosplenial cortex (RSG, ipsilateral) (Figure 5c) showed retrograde labeling exclusively after SCl injections.

If we take into account neuronal labeling generalized across the entire experimental cohort there was a separation of labeled RSD cells found after SCm injections and RSG after SCl injection, respectively. However, labeled RSG neurons were nevertheless found in two of the six SCm injection cases.

**Figure 6:**
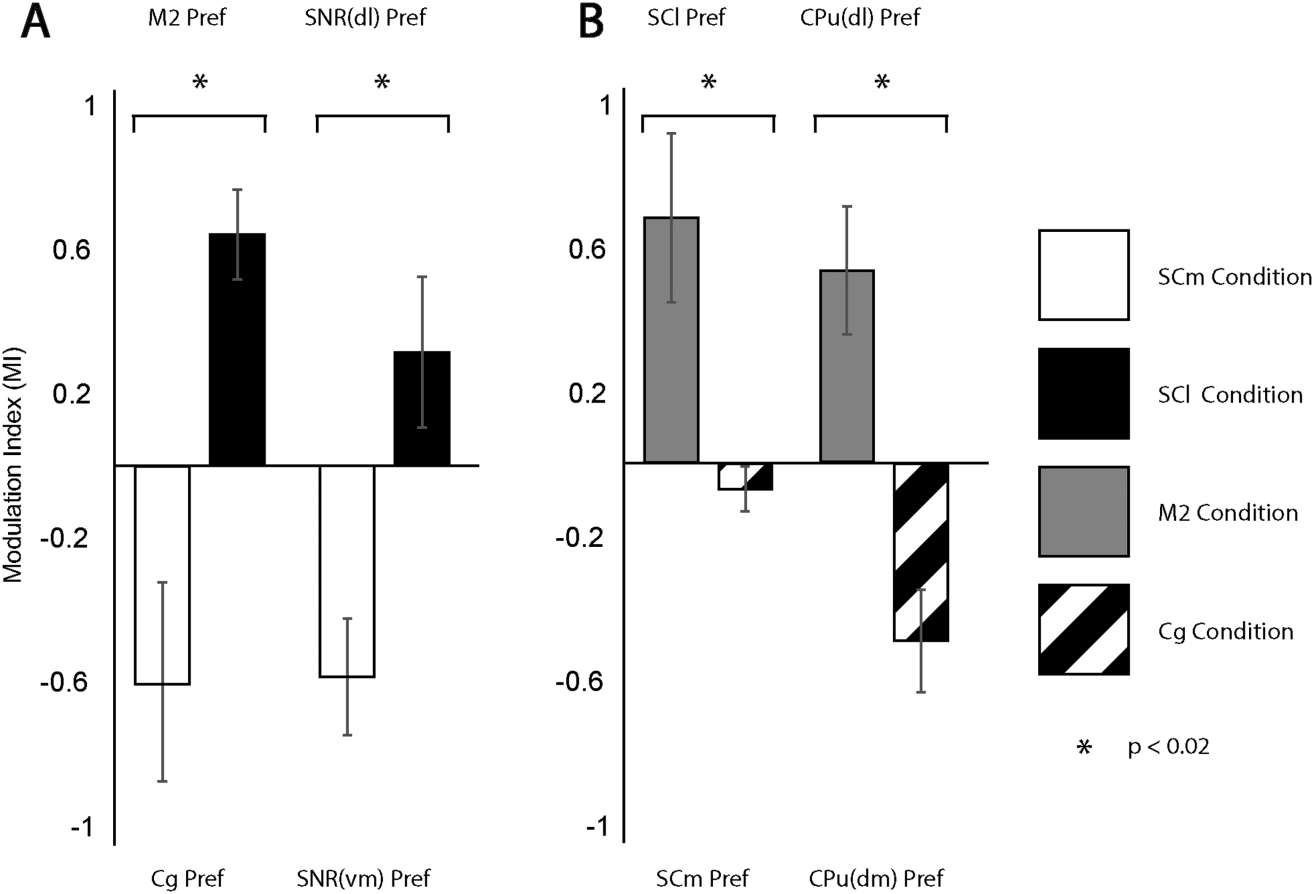
Modulation indices (MIs) for tracing data. (a) MIs of retrograde labeling in M2 versus Cg (left) and SNR(dl) versus SNR(vm) (right). (b) MIs of anterograde labeling in SCm versus SCl (left) and CPu(dm) versus CPu(dl) (right). White bars indicate MIs after SCm injections, black bars indicate MIs after SCl injections, gray bars indicate MIs after M2 injections, and dashed bars MIs after Cg injections) “*”represents *p* < .02

Retrogradely labeled cells after SCm and SCl injections were found in the M2 (ipsilateral), and in the Cg (ipsilateral). While these two areas showed retrogradely labeled cells after both, SCl and SCm injections, they did so to different degrees. The SCm injections resulted in higher numbers of labeled cells in the Cg (Figure 4d). Conversely, the SCl injections resulted in higher numbers of retrogradely labeled neurons in M2 (Figure 5d). This bias in connectivity for Cg and M2 was significant (*p* = .016, Mann–Whitney U test, Figure 6a left).

### 3.3 Retrograde labeling in the midbrain

Regions with retrogradely labeled cells only after SCm injections included the subthalamic nucleus (STh, ipsilateral), the dorsal raphe (DRV, bilateral), the external cortex of the inferior colliculus (ECIC, bilateral), the parabigeminal nucleus (PBG, bilateral), and the pontine nucleus (Pn, bilateral).

The prerubral field (PR, ipsilateral) showed retrogradely labeled cells exclusively after SCl injections. A number of midbrain regions contained retrogradely labeled neurons after injections of tracer into either subdivision of the SC. These included the lateral lemniscus (ll, ipsilateral), the PAG (bilateral), the mRt (bilateral), the substantia nigra (SNR, bilateral), and the SC (bilateral). The ll and the PAG showed similar density of retrogradely labeled cells, regardless of the injection site. The SC, mRt and SNR had differential numbers of retrogradely labeled cells following injection into the two subdivisions of the SC. The contralateral SCl was retrogradely labeled following injections into the SCm and the SCl. The mRt (ipsilateral) showed a higher number of retrogradely labeled cells after SCl than SCm injections. The SNR equally showed larger numbers of retrogradely labeled cells following SCl injection when compared to SCm injections. In addition, there was a significant (*p* = .016, Mann–Whitney U test) preference for the ventromedial SNR to show retrogradely labeled cells following SCm injections and for the dorsolateral SNR to show retrogradely labeled cells following SCl injections (Figures 4e, 5a, and 6a right).

### 3.4 Thalamic and hypothalamic areas

Retrogradely labeled cells after SCm, but not after SCl injections, were found in the lateral posterior thalamic nucleus, mediorostral part (LPMR, ipsilateral) and the ventromedial hypothalamic nucleus (VMH, ipsilateral).

SCl injections did not result in exclusive retrograde label in the thalamus or hypothalamus. A number of thalamic and hypothalamic regions contained retrogradely labeled neurons after both SCm, and SCl injections. The zona incerta ventral part (ZIV, ipsilateral) and dorsal part (ZID, ipsilateral) displayed retrograde neuronal labeling after injection into SCm and SCl. The ZIV was more strongly connected to the SC (l and m) than the ZID. Moreover, the neuronal projections from the ZI were spatially segregated, with the population projecting to the SCm being located in the dorsolateral region bordering on the dorsal lateral geniculate nucleus (DLG). The population projecting to the SCl was found in the ventromedial portion of ZI (Figures 4f and 5f).

### 3.5 Pretectum

The pretectal area (PT, ipsilateral) was retrogradely labeled only after SCm injections.

Retrogradely labeled cells were found in the ipsilateral nucleus of the posterior commissure (PCom, ipsilateral) after both SCm and SCl injections, while the contralateral PCom only sends efferents to the SCl.

**Figure 7:**
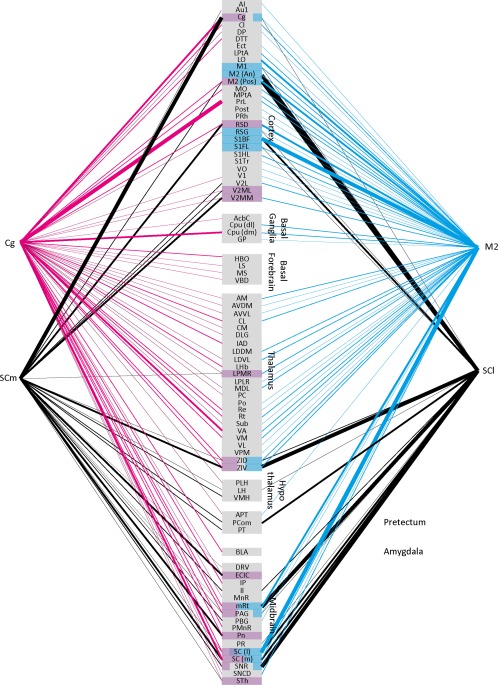
Connectivity matrix summary of SCm, SCl retrograde connections, and of Cg, and M2 anterograde connections. Connectivity is displayed in four levels, low, medium, high and very high, indicated by line thickness. Areas highlighted in colored boxes are those which receive input from the frontal cortex and also send projections to the relevant SC subdivision. Proportion of the box highlighted illustrates the strength of connection from the respective frontal area

To provide a general overview of input to the SC from the entire brain, we generated a connectivity diagram of the areas which exhibited retrogradely labeled cells after SCm and SCl injections, respectively, (Figure 7).

### 3.6 Anterograde tracing

**Figure 8:**
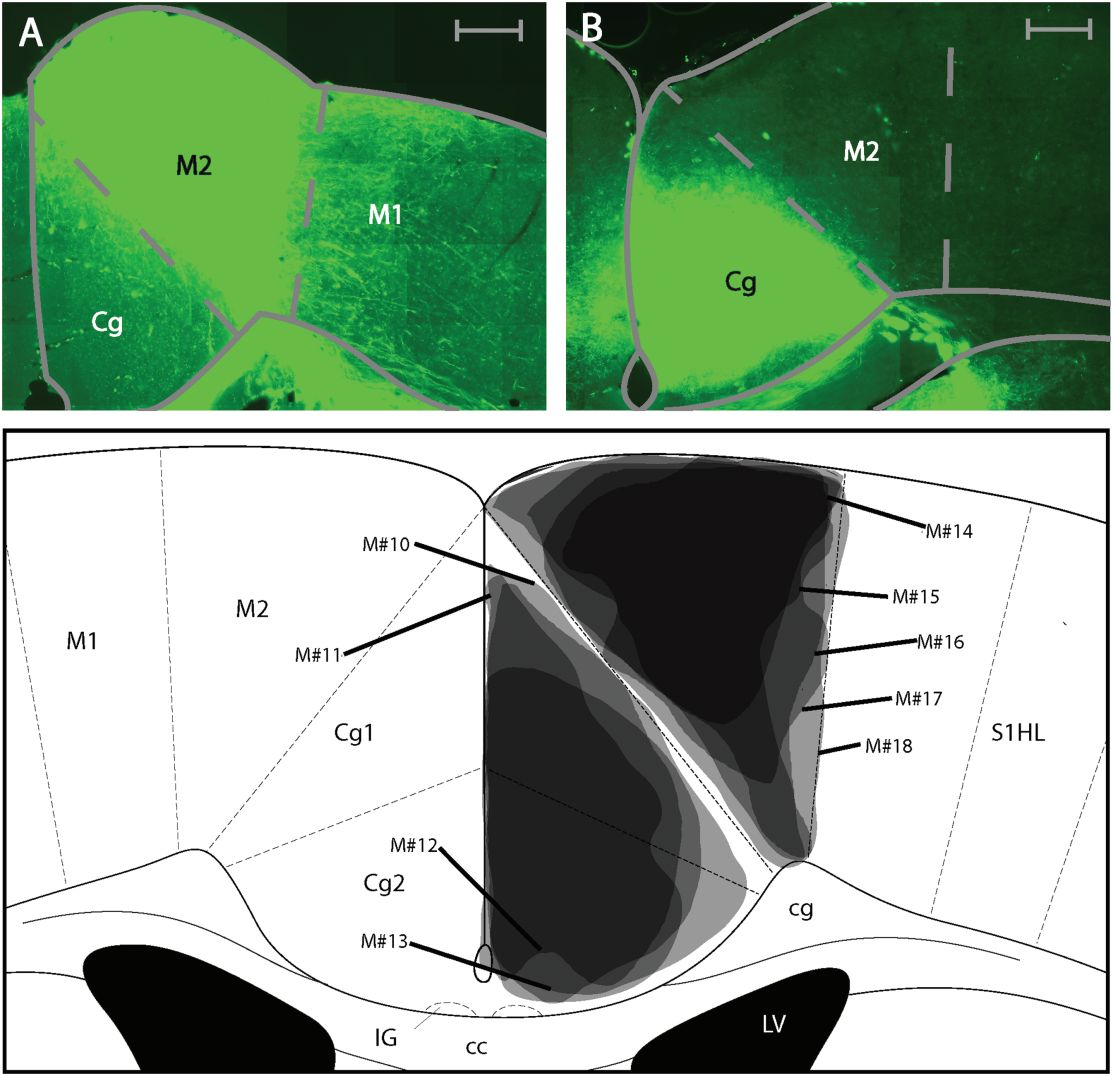
Injections sites for anterograde tracing. (a) Photomicrograph of biotinylated dextran anime injection into the M2. (b) Photomicrograph of biotinylated dextran amine injection into the Cg. All scale bars equate to 250 µm. (c) Summary of injection sites for all cases in the anterograde tracing in the Cg and M2. Each shaded area represents the extent of the labeled injection site for both the Cg and M2. The darker shading indicates overlap of injection volume

We performed five M2 and four Cg injections with the anterograde tracer BDA. The tracer in all cases was confined to the target area and did not leak into neighboring brain regions such as the corpus callosum (cc) and the third ventricle (Figure 8a–c). We will first describe cortical areas, where anterograde label was found exclusively after M2 injections, followed by a description of cortical areas where anterograde label was found exclusively after Cg injections. Thereafter, cortical areas will be described where anterograde label was found after both, M2 and Cg injections. This schema of description will be repeated for subcortical areas where anterograde label was found, focusing on areas where anterograde label was medium to strong. A complete list of all structures that showed anterograde label after M2 and Cg injections is given in Table 2. A connectivity matrix summary is displayed in Figure 7. Both regions predominantly projected ipsilateral, however a few regions also showed anterograde label contralateral to the injection site.

### 3.7 Cortex

**Figure 9:**
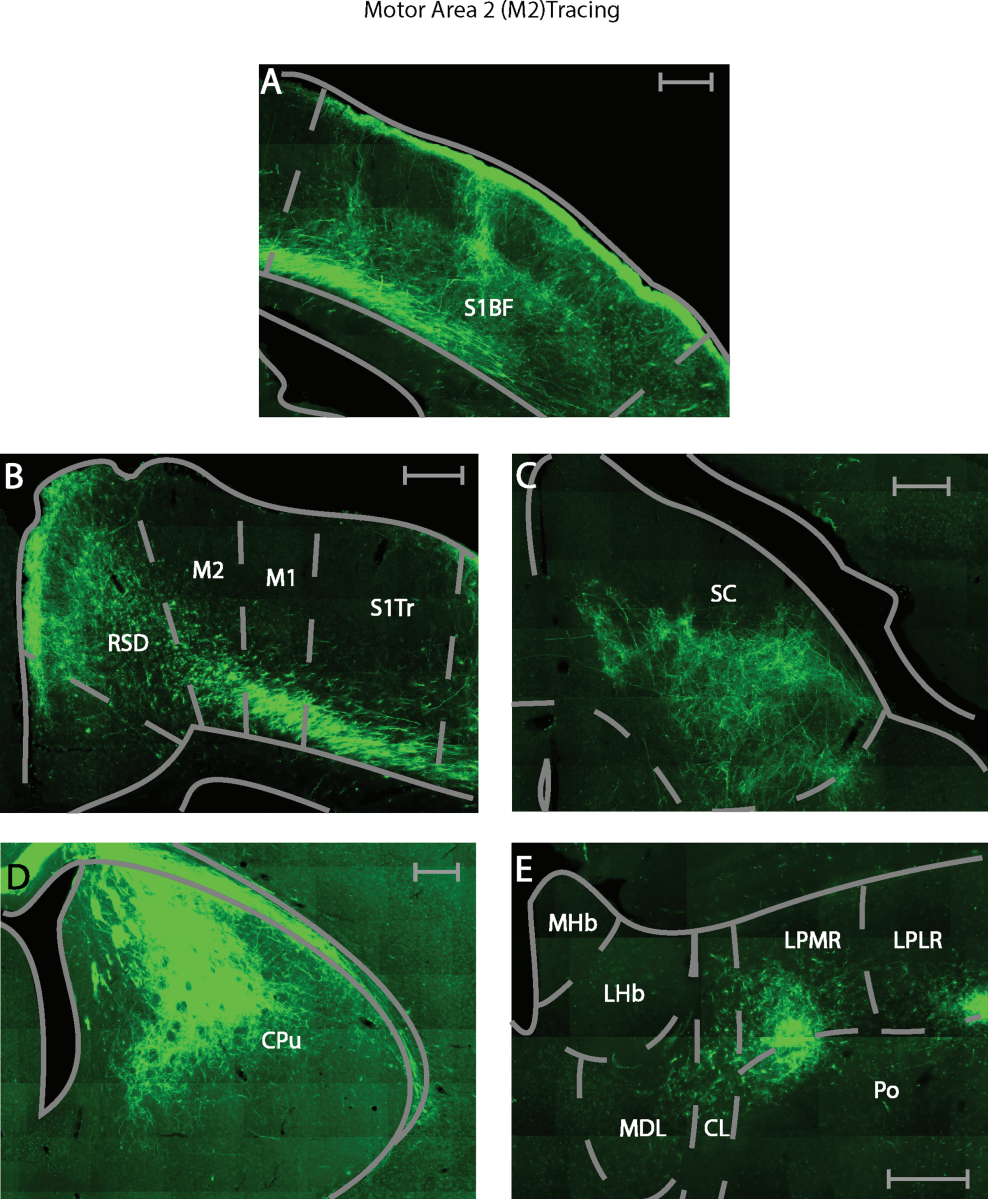
Example photomicrographs of anterogradely labeled brain areas after injection of BDA into the M2. (a) Labeling seen in the primary somatosensory area (S1BF). (b) Labeling seen throughout the RSD, M2, primary motor cortex (M1) and S1. (c) Labeling seen in the lateral portion of the superior colliculus (SCl). (d) Labeling seen in the dorsolateral striatum (CPu[dl]). (e) Labeling seen in the thalamus, namely the lateral posterior mediorostral and laterorostral part (LPLR, LPMR), the mediodorsal (MDL), the central lateral (CL) and the posterior (Po). All scale bars equate to 250 µm

The prefrontal cortex, the orbital cortex, lateral (LO, bilateral) and ventral (VO, bilateral) showed anterograde label exclusively after M2 injections. Anterograde label following M2 injections was found in virtually all primary somatosensory areas with stronger label in the barrel field (S1BF, ipsilateral) (Figure 9a), than the limb (S1FL, ipsilateral, S1HL, ipsilateral), or the trunk regions (S1Tr, ipsilateral, Figure 9b). A noticeable difference was found between the laminar connectivity profiles to S1BF and the rest of S1. In the S1BF anterograde labeling was concentrated in layers 1, 4, and 6, whereas for the other S1 regions, anterograde labeling was located in layers 5 and 6.

In addition the ipsilateral primary motor cortex (M1, ipsilateral, layers 1, 5, 6, Figures 8a and 9b), visual cortex V2L (ipsilateral across layers 1, 4 and 5), the parietal cortex (MPtA, ipsilateral, LPtA, ipsilateral, with preferential labeling in layers 5 and 6), the agranular insular cortex (AI, bilateral), the ectorhinal cortex (Ect, bilateral), postsubiculum (Post, ipsilateral), and the perirhinal cortex (PRh, bilateral) were anterogradely labeled exclusively after M2 injections.

**Figure 10:**
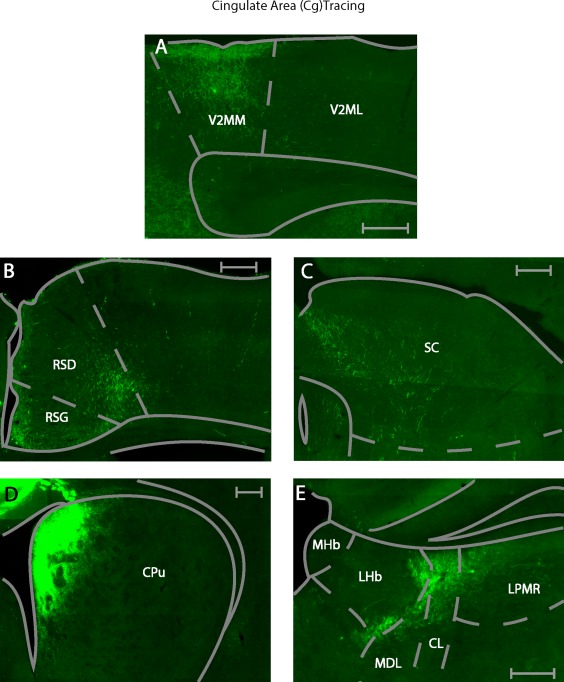
Example photomicrographs of anterogradely labeled brain areas after injection of BDA into the cingulate area. (a) Labeling seen in the secondary visual cortex (V2MM, V2ML). (b) Labeling seen throughout the RSD, RSG. (c) Labeling seen in the medial portion of the superior colliculus (SCm). (d) Labeling seen in the dorsomedial striatum (CPu[dm]). (e) Labeling seen in the thalamus, namely the LPMR, the MDL, the CL and the Po and the lateral habenula (LHb). All scale bars equate to 250 µm

Within the prefrontal cortex, the only area with exclusive anterograde labeling after Cg injections was the dorsal tenia tecta (DTT, ipsilateral). V2ML was the only sensory area with exclusive anterograde label after Cg injections (ipsilateral, Figure 10a across layers 1–5). In addition, the contralateral Cg showed anterograde label after Cg injections.

Cortical areas anterogradely labeled after injections into M2 and Cg included the dorsal peduncular cortex (DP, ipsilateral and biased toward the caudal end), the claustrum (Cl, bilateral, with a bias to the contralateral side), the primary visual cortex (V1, ipsilateral), the V2MM (ipsilateral), the prelimbic cortex (PrL, ipsilateral), the medial orbital cortex (MO, ipsilateral), RSD (ipsilateral, Figures 9b and 10b) and RSG, (ipsilateral, Figures 9b and 10b).

Despite the shared input of the above areas from Cg and M2, some biases or subregional differences were observed. PrL was more strongly connected to Cg than M2, ipsilaterally. M2 projected to more anterior locations in MO than Cg. Following M2 and Cg injections, the retrosplenial cortex showed anterograde label mostly in the RSD subdivision. This was stronger after M2 injections (compared to Cg injections). Moreover, M2 injections resulted in anterograde labeling in the upper layers of RSD (layers 1–3, Figure 9b), whereas the Cg injections resulted in anterograde label in the lower cortical layers of RSD (layers 5–6, Figure 10b). V2MM received more input from M2 than Cg.

### 3.8 Midbrain

All of the midbrain areas that received input from M2, also received input from Cg, while the opposite was not the case (see below).

Midbrain areas with anterograde label after Cg, but not M2 injections, were the ECIC, (ipsilateral), the STh (ipsilateral), the interpeduncular nucleus (IP, ipsilateral), the paramedian raphe nucleus (PMnR, ipsilateral), the median raphe nucleus (MnR, bilateral), and the Pn (ipsilateral).

Anterograde label in the midbrain after both M2 and Cg injections, was found in the cerebral peduncle (cp, ipsilateral), the SNR (ipsilateral), the substantia nigra pars compacta (SNC, ipsilateral), the dorsolateral and ventrolateral PAG (DLPAG, ipsilateral, VLPAG, ipsilateral), mRt (ipsilateral), the SCl (ipsilateral), and SCm (ipsilateral).

Despite the fact that the above areas showed anterograde label after either injection, some areas showed a spatial preference of anterograde labeling within their subdivisions. The PAG was more strongly labeled in the dorso‐lateral part (DLPAG) after Cg injections, while it was more strongly labeled in the ventro‐lateral part (VLPAG) following M2 injections. The substantia nigra, while receiving input from both areas, did so in a topographically biased manner. The SNR received connections from both the Cg and M2 which terminated onto the ventromedial part of the area. The SNC received sparse connections from the Cg and more abundant connections from M2.

Other midbrain regions received stronger input from one of the two areas. The mRt showed more anterograde label after M2 than after Cg injections. The SCl showed more anterograde label than SCm after M2 injections, whilst the opposite was the case after Cg injections (Figures 9c and 10c). This preference was significant (*p* = .016) (Figure 6b left). Additionally, anterograde label from the Cg was found in more anterior parts of the SC than that arising from M2.

### 3.9 Basal forebrain

The basal forebrain did not show anterograde label after M2 injections. Anterograde label was found in parts of the medial basal forebrain after Cg injections. Specifically, the medial septal nuclei (MS, bilateral), the lateral septal nuclei (LS, bilateral), the diagonal band, vertical limb (VDB, bilateral), and the diagonal band, horizontal limb (HDB, bilateral) showed anterograde label. The HDB connections expressed a bias for ipsilateral over contralateral connectivity.

### 3.10 Basal ganglia

The globus pallidus (GP, ipsilateral) was anterogradely labeled only after M2, not after Cg injections. The core of the nucleus accumbens (AcbC, ipsilateral) received low levels of input from Cg, but no input from M2.

The striatum showed anterograde label after either M2 or Cg injections, albeit in a topographically segregated manner. The dorsolateral striatum (CPu[dl], ipsilateral) was more strongly labeled after M2 injections. Conversely, the dorsomedial striatum (CPu[dm], ipsilateral) was more strongly labeled following Cg injections (Figures 9d and 10d). This topographical difference was significant (*p* = .016, Mann–Whitney U test) (Figure 6b right). Contralaterally, the CPu(dl) received few projections from M2, while the CPu(dm) received few projections from the Cg.

### 3.11 Thalamic and hypothalamic areas

Anterograde labeling was observed only after M2 injections in the lateral posterior thalamic nucleus, laterorostral part (LPLR, ipsilateral, Figure 9e), the dorsal portion of the posterior thalamic nuclear group (Po, ipsilateral, Figure 9e), the laterodorsal thalamic nucleus, dorsomedial part (LDDM, ipsilateral), and the ventrolateral thalamic nucleus (VL, ipsilateral, dorsal portion).

The Cg projects to a larger number of thalamic nuclei, which were not matched by projections from M2. Exclusive anterograde label following Cg injections was found in the paracentral thalamic nuclei (PC, ipsilateral), the central medial thalamic nuclei (CM, bilateral), and the lateral habenular nucleus (LHb, ipsilateral, Figure 10e). Projections from Cg targeted the interanterodorsal thalamus (IAD, bilateral), with an ipsilateral bias. Cg projections to the dorsal lateral geniculate nucleus (DLG, ipsilateral) were found in the dorsolateral part of the area. Selective projections to the hypothalamus were restricted to the peduncular part of the lateral hypothalamus (PLH, ipsilateral).

Areas with anterograde label after both, M2 and Cg injections included the anteroventral thalamus, dorsomedial (AVDM, ipsilateral) and ventrolateral (AVVL, ipsilateral), the submedius thalamic nucleus (Sub, ipsilateral), the reticular nucleus (Rt, ipsilateral), the zona incerta, dorsal (ZID, ipsilateral) and ventral (ZIV, ipsilateral) portions, the ventromedial thalamic nucleus (VM, ipsilateral), the central lateral nucleus (CL, ipsilateral, Figures 9e and 10e), anteromedial thalamic nucleus (AM, ipsilateral), the laterodorsal thalamic nucleus, ventrolateral part (LDVL, ipsilateral), the mediodorsal thalamic nucleus, lateral part (MDL, ipsilateral), and the lateral posterior thalamic nucleus, mediorostral part (LPMR, ipsilateral, Figures 9e and 10e), the ventral anterior thalamic nucleus (VA, ipsilateral), and the reuniens thalamus (Re, bilateral).

A few thalamic areas showed partial topographical label segregation after M2 and Cg injections. In VM, anterograde label following Cg injections occurred throughout the area, whereas anterograde label following M2 injections was restricted to the ventral region. In CL, anterograde label following Cg injections was restricted to the dorsal portion of the area, while input from the M2 was found further down the dorsal–ventral axis (Figures 9e and 10e).

In addition, anterograde label strength in some areas differed depending on the injection site. The AM, LDVL, MDL, and the LPMR showed more anterograde label after M2, than after Cg injections (Figures 9e and 10e). All of these areas displayed a topographical preference in their labeling pattern. Label in AM, regardless of injection site (M2, Cg), was found in the lateral part. Label in LDVL after M2 injections was found more in the ventral part; whereas no preference was found following Cg injections. M2 injections resulted in preferential anterograde label in the lateral portion of the MDL, while Cg injections resulted in preferential anterograde label in the dorsal portion of MDL. M2 originating label in LPMR occurred more ventromedially, while Cg originating label occurred more dorsomedially (Figures 9e and 10e). The Cg projected more heavily to VA and Re, than M2 did.

### 3.12 Amygdala

Anterograde label was found in the basolateral amygdaloid nucleus, anterior part (BLA, ipsilateral) following Cg injections, but not M2 injections.

### 3.13 Pretectum

The anterior pretectal nucleus (APT, ipsilateral) showed anterograde label following Cg and M2 injections.

## 4 Discussion

We delineated the main cortical and subcortical inputs to the medial and lateral SC of the mouse, as well as the target areas of two key frontal areas providing strong preferential input to these SC subdivisions.

We found limited overlap in the cortical and subcortical afferents to the SCm and SCl. The majority of regions which project to the SCm have visual, extra‐personal (far) space and negative affective state related functionality. The majority of regions which project to the SCl have somato‐motor, peri‐personal (near) space related functionality. Areas which were labeled after injection into either of the two subdivisions of the SC, often showed topographically segregated cell populations with limited spatial overlap.

The main prefrontal areas providing segregated inputs to middle and lower layers of the SC, Cg, and M2, equally target functionally segregated networks. Areas which received input solely from the Cg are functionally related to vision, emotional state and avoidance behaviors. Areas which received input solely from M2 are functionally related to somato‐sensation, gustation, and approach behaviors. Areas which received projections from both Cg and M2 often had a tendency to have topographical segregation, suggesting that functional specialization in these areas exists at the level of subpopulations.

### 4.1 Relations to previous literature

#### 4.1.1 SC retrograde tracing

Our retrograde tracing data are largely consistent with the existing literature (Taylor, Jeffery, & Lieberman, 1986). However, the differential connectivity between the SCm and SCl, while largely in agreement with the respective analysis in the rat (Comoli et al., 2012), also shows some discrepancies. Additional discrepancies exist when compared to the mouse whole brain imaging project (Oh et al., 2014).

Comoli et al. (2012) reported retrograde labeling in the ectorhinal, infralimbic, prelimbic cortices, the parietal region, the temporal association area (TEa), the postsubiculum, the premamillary nucleus, and the LGN after injections into the SCm, which we did not find. Following SCl injections, retrograde label was not found in the insular cortex in our study, while it was reported by Comoli et al. (2012). Some of these discrepancies can be resolved. For example the parietal region uncovered to project to SCm by Comoli et al. (2012), is likely to be equivalent to the region termed the secondary visual cortex in our work, a consequence of the sometimes variable use of nomenclature in relation to mouse cortical areas (Guo et al., 2014; Harvey, Coen, & Tank, 2012). In addition, we found retrogradely labeled cells in areas, which were not reported by Comoli et al. (2012). These included the ECIC, the PBG, the Pn and the prerubral field. The input from the PBG and the ECIC to the rat SC, however, has been shown previously (Taylor et al., 1986). The differences observed between the results presented here and the Comoli paper may reflect species specific connectivity and/or differences in relative injection site.

Oh et al. (2014) reported retrogradely labeled cells in a variety of regions which were not labeled in our data. These included projections to both the SCm and SCl from the prefrontal orbital cortex, primary sensory areas such as the AuD, thalamic and hypothalamic areas (LGN, Po, VM, anterior hypothalamic nucleus, dorsomedial nucleus of the hypothalamus (DMH), posterior hypothalamic nucleus, parafascicular nucleus), the amygdala, and the midbrain (the mammillary nucleus, pedunculopontine nucleus, ventral tegemental area (VTA), red nucleus).

Furthermore, their data uncovered areas which connected solely to the SCm, which were not found in our results, such as the prefrontal area IL, primary sensory areas (V1, S1), temporal cortical areas (Ect, TEa, postrhinal area, subiculum, postsubiculum), the amygdala and the hippocampus.

Brain regions found to connect only to the SCl in the Oh et al. (2014) paper, but not in our data, included prefrontal (AI), sensory (V2, S2), thalamus and hypothalamus (MDL, VPM, arcuate hypothalamic nucleus, VMH), and the midbrain (anterior pretectal nucleus, intermediate reticular nucleus, Pn, DRV) (Oh et al., 2014).

### 4.2 M2/Cg anterograde tracing

In general, the projections identified from Cg and M2 mouse cortical and subcortical targets are similar to those found previously in the rat (Domesick, 1969; Gabbott, Warner, Jays, Salway, & Busby, 2005; Kamishina, Conte, Patel, Tai, Corwin, & Reep 2009; Reep, Corwin, Hashimoto, & Watson, 1987; Vogt & Miller, 1983). However, following M2 injections we did not find anterograde labeling in the PC, the STh, and the dorsal raphe nucleus, unlike previous reports. Moreover, we found anterograde label in the SNC and the AV after M2 injections, which were not reported in previous studies in the rat. Again, these difference may be species specific, or could result from differences in injection sites and labeling techniques.

However, in comparison with more recent brain mapping studies, some discrepancies were found (Oh et al., 2014; Zingg et al., 2014). For example, a number of areas targeted by M2 and by Cg were found by Oh et al. (2014), as well as Zingg et al. (2014), which were not uncovered in our results. These included the frontal pole, the sensory related area AuD, the piriform cortex, the substantia innominata, some areas within the thalamus and hypothalamus (AD, paraventricular thalamic area, DMH, preoptic area), and within the midbrain (mammillary nucleus, VTA, central raphe nucleus).

Following injections into Cg, Oh et al. (2014) found projections to prefrontal areas (AI, IL, orbital), primary sensory areas (M1), cortical areas (entorhinal cortex, ECT, TEa, endopiriform cortex, POST), the thalamus and hypothalamus (Po, anterior hypothalamic nucleus, paraventricular hypothalamus) the midbrain (pretectal nucleus, PCom), and the hippocampus. Our injections did not show label in these areas.

Additionally, following injection into M2, Oh et al. (2014) reported anterograde connections with the gustatory region, the perirhinal cortex, the parafascicular thalamic nucleus, the AbC, the midbrain (APT, PBG, tegmental reticular nucleus) and the amygdala, which we equally did not find.

### 4.3 Relation of anatomical visual connectivity to functionally defined visual regions

We have identified segregated connectivity pattern from secondary visual areas onto the SC, and from the prefrontal areas (Cg, M2) to those secondary visual cortical areas. Due to the increased focus in the literature on functionally defined areas it is important to relate anatomically defined label to these functional terms (Garrett, Nauhaus, Marshel, & Callaway, 2014; Marshel, Garrett, Nauhaus, & Callaway, 2011; Wang & Burkhalter, 2007).

In the SCm cohort, labeling in the secondary visual cortex was found in all parts. Anatomically defined secondary visual cortex would correspond to a number of functionally defined visual regions, specifically the anteromedial area (AM), rostrolateral area (RL), and posteromedial area (PM) (Wang & Burkhalter, 2007). AM has a high temporal frequency preference which may aid an animal in detecting fast moving stimuli such as predators (Marshel et al., 2011). PM has a comparatively high spatial frequency preference which may aid in object identification in the visual environment. Furthermore, the more medial parts of AM and PM have been shown to respond to stimuli in the peripheral visual field (Garrett et al., 2014; Marshel et al., 2011). Similarly, the visual projections of Cg terminate in V2MM and V2ML, which may match the functionally defined areas AM and PM. Thus, AM and PM would receive innervation from Cg, which provide the SCm with information regarding the location and spatial features of visual stimuli in the upper/peripheral visual field. This circuit may prime avoidance behaviors when faced by potential predators.

The visual projections from M2 terminate in the V2L region, which, as defined in this study, may match the functionally defined laterointermediate area (LI), rostrolateral area (RL), and PM (Wang & Burkhalter, 2007). LI, similarly to PM, has a higher spatial frequency preference than other higher visual areas and may be related to object recognition/classification. The functional region RL has been previously assigned to be part of the parietal cortex of the mouse and has been implicated in visual and whisker multisensory integration (Olcese, Iurilli, & Medini, 2013). RL has a preference for high temporal frequency stimuli and represents the lower central visual field (Garrett et al., 2014; Marshel et al., 2011). In conjunction with our data, this suggests that M2 connections to RL may enhance processing of visual information in the lower visual field to aid orienting/approach behaviors.

### 4.4 Functional implications

#### 4.4.1 SCm and avoidance behaviors

The SCm contains a retinotopic map of the upper visual space, via projections from the retina, primary, and secondary visual areas (V1, V2MM, V2ML, V2L) (Ahmadlou & Heimel, 2015). Looming stimuli in the upper visual field elicits fear responses that are mediated from the SC through the LP to the amygdala (Wei et al., 2015). Furthermore, optogenetic stimulation of SCm elicits avoidance behaviors which are initiated via the PBG and the Pn (Shang et al., 2015). Reciprocal connectivity to the SCm from LP, a possible rodent homologue of the pulvinar, may deliver information to guide orienting behaviors (Wei et al., 2015). Finally, areas directly involved in fear processing such as the VMH and the PAG may conduct fear‐state information to the SC (Dielenberg, Hunt, & McGregor, 2001). Once the avoidance sensorimotor transduction has been processed in the SCm, signals can be sent through the uncrossed tecto‐reticulo‐spinal tract which mediates the avoidance related motor output (Redgrave, Dean, Mitchell, & Odekunle, 1988).

#### 4.4.2 SCl and approach behaviors

The SCl is retinotopically mapped to the lower visual space, where appetitive stimuli, such as prey or offspring are likely to occur, both of which require approach‐orienting responses, (Ahmadlou & Heimel, 2015). In rats, appetitive hunting and whisking behavior results in increased c‐FOS expression within the SCl, and lesions of the SCl decrease predatory orienting behaviors (Favaro et al., 2011; Furigo et al., 2010). Research groups who investigate auditory or odor cued orienting responses in the SC often place probes (electrodes, optrodes) in the lateral portion of the SC (Duan, Erlich, & Brody, 2015; Felsen & Mainen, 2012; Stubblefield, Costabile, & Felsen, 2013), and thus our knowledge regarding stimulus processing in the mouse SC might be biased toward appetitive stimulus types. Once processed, the SCl sends the information through the crossed tecto‐reticulo‐spinal tract to brain stem motor nuclei to initiate approach behavior (Redgrave, Dean, & Westby, 1990).

Although we have highlighted an existing dichotomy in the separation of approach and avoidance behaviors regarding the location of stimuli in the visual field, it must be noted that this segregation is not complete. Studies have used visually stimuli in the upper visual field which require approach behaviors (Harvey, Collman, Dombeck, & Tank, 2009; Scott, Constantinople, Erlich, Tank, & Brody, 2015). Conversely, other studies have employed stimuli which occur in the lower visual field, and which require avoidance behaviors (Ho et al., 2015; Manita et al., 2015). However, in these studies the stimuli have usually been presented a large number of times and have been associated with either a positive or negative outcome. This associative learning may then override the innate visual field associated orienting biases that are predominantly present. Alternatively, the bias described in this study, may be subject to context dependent modulation, such that it can be suppressed and even reversed if circumstances so dictate.

#### 4.4.3 Cortical control of orienting behavior

M2 and Cg innervate different sections of the SC. This suggests that they control separate types of orienting behavior. If so, it should be reflected in their cortical and subcortical efferent projections. We investigated this by anterograde tract tracing, and indeed uncovered a difference in projection patterns.

M2 mostly sends efferents to SCl and somatosensory cortical areas. M2 in the mouse may be the homolog to FOF in rats (Erlich et al., 2011). Behaviorally, M2 has been implicated in top–down modulation of somatosensory based orienting and appetitive approach behaviors (Erlich et al., 2011; Guo et al., 2014). Additionally, M2 projects to parietal regions (MPtA, LPtA), which are involved in evidence accumulation and decision formation (Hanks et al., 2015). M2 neurons encode a categorical classification of evidence in decision making, while parietal neurons encode a more continuous representation of accumulated evidence (Hanks et al., 2015). The connection from M2 to MPtA and LPtA suggests that parietal cortex and frontal cortex interact in a reciprocal manner, rather than in a simple feed‐forward scheme where accumulated evidence in one area is converted into a categorical representation at a higher level. Lesions of M2 in rats cause a deficit in orienting, while microstimulation elicits orienting type behaviors (Cowey & Bozek, 1974; Sinnamon & Galer, 1984). A recent study has indicated that both the M2 and the SCl are involved in the generation of short term memory representations that are required for sensory orienting (Kopec, Erlich, Brunton, Deisseroth, & Brody, 2015). Taken together this information lends weight to the role of the M2 area in guiding orienting approach related behaviors which are mediated via the SCl.

The Cg is the major source of prefrontal input into the intermediate and lower layers of the SCm. Behaviorally, it has been implicated in top–down modulation of aversion related behaviors. Lesions of the Cg in rabbits reduces avoidance behaviors in relation to noxious stimuli (Gabriel, Kubota, Sparenborg, Straube, & Vogt, 1991). Furthermore, Cg activity can precede aversion responses to pain (Freeman, Cuppernell, Flannery, & Gabriel, 1996). Indeed, stimulation of Cg in rodents facilitates nociceptive reflexes (Calejesan, Kim, & Zhuo, 2000). The Cg is heavily interconnected with regions involved in pain and fear processing (MD, amygdala, and hypothalamus). Cg projects to a number of areas in the basal forebrain which are part of the arousal/attention network. Activation of the Cg could thus result in heightened states of arousal, through activation of those pathways. Taken together this indicates a role of the Cg in pain and fear processing, which would result in the planning of avoidance behaviors, and which can be mediated via the SCm.

In conclusion, our study has revealed anatomically segregated circuits in the mouse brain that likely orchestrate approach and avoidance behavior, respectively. Avoidance behavior is likely subserved by Cg, secondary visual cortices, auditory areas, and the dysgranular retrospenial cortex in conjunction with SCm. Conversely, approach/appetitive behaviors is likely sub‐served by M2, somatosensory cortex, and the granular retrospenial cortex in conjunction with the SCl.

## Conflict of interest

The authors declare they have no competing financial interests.
